# Notes and News

**Published:** 1899-09-16

**Authors:** 


### Notes and News.

(Collected and Communicated.)

At the special court held at the London Hospital last week Mr. Edward Raphael's gift of £10,000 was announced towards the endowment of a new Jewish ward; and it was reported that the gift of £60,000 by the late Baroness de Stern towards a convalescent home would be appropriated by spending £10,000 upon the site, and £50,000 be placed to the maintenance of the institution.

In the discussion on vaccination at Portsmouth a point was made of the slowness with which the glycerinated lymph dries, as very materially complicating the performance of the operation. One cannot wait three-quarters of an hour at each case. Yet if one puts on an absorbent pad it is a question whether it does not suck up the lymph, and so spoil the result; while if one leaves the application of the pad to the mother one does but tempt misfortune. Indeed, it may be doubted whether such a course complies with the instructions contained in the Order as to protecting the vaccinated surface against extraneous infection. The more one considers the responsibilities of the public vaccinators the smaller do their fees appear.

In summing up the work performed during the past three months by the ladies who have been appointed in Birmingham as "health visitors," to inspect the homes of the poor and to give advice on health subjects, Dr. Hills, the medical officer of health, shows that much useful work has been done in urging the necessity for ventilation, it being found in many instances that not only were the bedroom windows left unopened, but that the chimneys were stopped up. The curious indifference of the poor to the advantages of pure air was shown in another way, which deserves the special attention of those who are concerned in calculating the cubic space of dwelling-rooms. It was not uncommon to find that notwithstanding the over-crowded condition of the bedrooms the whole accommodation which the houses provided was not utilised. In fact, in several cases it was discovered that the whole family slept in one room while a second room was left vacant. Thus does the habit of accepting an inferior standard of living perpetuate insanitary evils even when necessity no longer compels people to submit to them.

At the Sanitary Congress at Southampton attention was drawn by the medical officer of Liverpool to the danger of inefficient or no vaccination at all amongst the unskilled labourers who frequent the docks at Liverpool. Any epidemic amongst them, he pointed out, was liable to seriously injure trade, seeing that foreign Consuls made strict inquiry as to infected ports.

The fact that the mosquito is concerned in the dissemination of malaria has stimulated research in all malarial districts within the reach of scientists. The practical question is, of course, the extermination of the insect. In and around Rome especial opportunities for useful experiments exist, and two scientific men—Professor A. Celli and Dr. Casagrandi—have made studies in this direction. Of course, different methods are necessary in dealing with the different stages in the development of the insect, but the destruction of the perfect mosquito is that which will occupy the attention of the public primarily; the destruction of the insect in its larval stage seems, however, the most hopeful method.

The *Practitioner* makes very merry over the proceedings at the Portsmouth meeting of the British Medical Association. In describing the general meetings, which, it says, have degenerated from comedy to farce, it describes how "butter is ladled profusely over the President of the Association," how the officers "congratulate each other on the labours which they have lavished on their arduous duties—mostly performed by others," and how "in accordance with the general way of this best of all possible worlds . . . the one officer of the Association who has no share in the butyraceous honours so freely bestowed is the Editor of the *British Medical Journal.*" He, it says, is the Atlas on whose shoulders rests the world of the Association; "but one notes with regret, though hardly with surprise, that there are no compliments for him." The whole thing, says the *Practitioner*, is a pleasing illustration of what Mr. Pecksniff called "human nature."

A letter appeared in Tuesday's *Times* on the subject of secret commissions, which, although it was merely signed "F.R.S.," we think we may fairly attribute to the pen of the Regius Professor of Physic at Cambridge, vindicating the medical profession from the aspersions thrown upon it, and on Wednesday a further letter appeared from Sir Edward Fry, apparently replying on the whole case. In it he says: "Some of my critics insist that I have brought an accusation against the medical profession at large, and that in spite of my explicit statement to the contrary in my letter. Why some white sheep of the profession should be so offended when the black sheep are spoken of is not very easy to understand." But this is the very point. If he had said "black sheep" at first, no one would have been offended. Even "F.R.S.," who champions the profession at large, says he "cannot tell what corruption may possibly exist upon the skirts of the medical as of the legal profession." We should indeed be sorry to have to defend our black sheep, but when Sir Edward Fry referred to the "lower walks of the profession" we doubt whether many people read him as alluding only to "black sheep."

